# End-to-end pseudonymization of fine-tuned clinical BERT models: Privacy preservation with maintained data utility

**DOI:** 10.1186/s12911-024-02546-8

**Published:** 2024-06-12

**Authors:** Thomas Vakili, Aron Henriksson, Hercules Dalianis

**Affiliations:** Department of Computer and Systems Sciences, Stockholm University, P.O. Box 7003, 164 07 Kista, Stockholm Sweden

**Keywords:** Natural language processing, Language models, BERT, Electronic health records, Clinical text, De-identification, Pseudonymization, Privacy preservation, Swedish

## Abstract

Many state-of-the-art results in natural language processing (NLP) rely on large pre-trained language models (PLMs). These models consist of large amounts of parameters that are tuned using vast amounts of training data. These factors cause the models to memorize parts of their training data, making them vulnerable to various privacy attacks. This is cause for concern, especially when these models are applied in the clinical domain, where data are very sensitive.

Training data pseudonymization is a privacy-preserving technique that aims to mitigate these problems. This technique automatically identifies and replaces sensitive entities with realistic but non-sensitive surrogates. Pseudonymization has yielded promising results in previous studies. However, no previous study has applied pseudonymization to both the pre-training data of PLMs and the fine-tuning data used to solve clinical NLP tasks.

This study evaluates the effects on the predictive performance of end-to-end pseudonymization of Swedish clinical BERT models fine-tuned for five clinical NLP tasks. A large number of statistical tests are performed, revealing minimal harm to performance when using pseudonymized fine-tuning data. The results also find no deterioration from end-to-end pseudonymization of pre-training and fine-tuning data. These results demonstrate that pseudonymizing training data to reduce privacy risks can be done without harming data utility for training PLMs.

**Supplementary Information:**

The online version contains supplementary material available at 10.1186/s12911-024-02546-8.

## Introduction

The popularization of the transformer architecture [1] in the past few years has led to rapid advances in natural language processing (NLP). Many benchmarks are now dominated by pre-trained language models (PLMs) that learn to model language using unlabeled corpora. There are many PLM architectures, and this article focuses on the BERT architecture [2], which is widely used and competitive in many NLP benchmarks. PLMs typically consist of hundreds of millions, even billions, of parameters which are trained on enormous amounts of unlabeled training data. The sizes of the corpora used to pre-train these models are typically in the range of tens of gigabytes or even terabytes of data. The BERT models used in this study consist of over 100 million parameters and are pre-trained on around 6 billion tokens [2, 3]. On the other end of the scale, the largest publicly available version of Llama 2 consists of 70 billion parameters tuned using a corpus spanning 2 trillion tokens [4].

PLMs have shown great promise in several NLP domains, and the clinical domain is no exception. State-of-the-art results in clinical NLP tend to rely on PLMs, e.g., for temporal relation extraction [5], text similarity [6], concept normalization [7], adverse drug event extraction [8], medication event extraction [9] and information extraction [10]. However, while PLMs are generally pre-trained using readily available corpora in the general domain – e.g., Wikipedia and other data sources on the Internet – research suggests that using generic PLMs in highly specialized domains such as healthcare may be suboptimal due to significant domain differences [11, 12], even in the presence of large language models like T5-XL and GPT-3 [13]. This has motivated efforts to develop domain-specific clinical PLMs. There are different approaches to developing domain-specific PLMs [14], including pre-training a new language model from scratch with in-domain data, e.g., in the form of clinical text from electronic health records (EHRs). An alternative approach is to adapt an existing, generic PLM to the target domain by continuing to pre-train it with in-domain data. The vocabulary of the model can be retained or adapted to account for domain differences. This continued pre-training is known as *domain-adaptive pre-training* [15–17].

While PLMs have shown great promise in solving important NLP problems, their reliance on increasingly large numbers of parameters and vast corpora causes models to memorize parts of their training data [18–20]. This tendency is undesirable in many use cases but also has important implications for privacy. When models are domain-adapted using clinical data, these privacy risks must be mitigated. Clinical data often describes sensitive information that must be protected, not just for ethical reasons but also due to current regulations.

One way to reduce the privacy risks of using clinical data is to remove sensitive information. An important technique for doing so is called *pseudonymization*, which involves locating sensitive passages using named entity recognition (NER) and substituting them with realistic surrogate data. This technique has been applied to data for pre-training language models [21, 22] and for fine-tuning models [20, 23], with successful results. However, previous research has only studied these two training steps in isolation.

In this paper, we demonstrate the first example of a clinical language model that has been *fully pseudonymized* in both the domain-adaptive pre-training and fine-tuning steps. This is done by:

- Pseudonymizing datasets for five clinical downstream tasks.
- Fine-tuning and evaluating a total of 300 models through 10-fold cross-validation of 30 different combinations of pseudonymized data and models.
- Comparing all models in terms of F_1_ to determine if any statistically significant differences in predictive performance exist.

The results show that end-to-end pseudonymization can be successfully applied to the pre-training and fine-tuning of language models. We find that end-to-end pseudonymization preserves privacy and simultaneously retains the utility of the data for domain-adaptive pre-training and fine-tuning of PLMs.

## Background

This study focuses on mitigating the privacy issues of modern transformer models in NLP using pseudonymization. This section gives a more detailed motivation for how these models are vulnerable to privacy attacks and why pseudonymization is a good privacy-preserving technique. Other privacy-preserving techniques are discussed, and previous works on pseudonymization are presented to provide the context in which this study is situated.

### Privacy attacks

As mentioned in the introduction, large language models can be susceptible to privacy attacks. This susceptibility is partially due to the self-supervised pre-training objectives that tend to involve reconstructing a noisy or obscured version of the training data. For example, BERT models are pre-trained using *masked language modeling* [2], which involves reconstructing a version of the training data in which some tokens have been replaced with [MASK] tokens. The pre-training is then performed using large text corpora with unknown quantities of sensitive information, and the learned features are encoded in millions or billions of parameters.

Privacy attacks targeting PLMs aim to extract information about their training corpora. The attacks do so by targeting the information encoded in the parameters of the models. Depending on the objective, these attacks can be categorized into two main classes. *Training data extraction attacks* aim to reconstruct data used to train a model. This is a severe form of attack since it can result in the disclosure of sensitive information about persons described in the training data of a model. In the clinical domain, this could mean exposing the details of a patient’s medical history. Training data extraction attacks require an effective algorithm for sampling information from a model; however, such algorithms are not (yet) described for all models [24–27]. Nevertheless, there are examples of successful training data extraction attacks targeting generative systems such as GPT-2 and ChatGPT [19, 28].

*Membership inference attacks* aim to discern whether or not a particular datapoint has been used to train a target model [29]. In a clinical context, this information could reveal if a patient associated with an EHR has visited a set of clinical units associated with particular health problems. This category of attacks typically involves measuring the *reaction* of a model to a set of datapoints and using this information to distinguish between members and non-members of the training data [30, 31]. Successful membership inference attacks may pose a privacy threat in themselves, but are also often used as a building block in training data extraction attacks when determining whether the algorithm has extracted a real or spurious datapoint.

### Privacy-preserving techniques

Several privacy-preserving techniques have been developed to mitigate the privacy threats described in the previous section. In this section, a non-exhaustive list of techniques will be described to provide context for why this study focuses on the pseudonymization of training data. Other promising and oft-mentioned techniques include differential privacy, homomorphic encryption, and synthetic training data.

Differential privacy is a notion of privacy that was originally designed for database records. The idea is that, given a datapoint *d* and two datasets *D* and $D'$ differing only in that $d \in D$ while $d \notin D'$, the output of any aggregation of these datasets should be close to indistinguishable [32]. As it is typically formulated, we have $(\epsilon ,\delta )$-differential privacy [33] for an aggregation *M* with range *R* if

$$\begin{aligned} P[M(D) \in R] \le e^\epsilon P[M(D') \in R] + \delta . \end{aligned}$$

Differential privacy has also been adapted for deep learning. The DP-SGD algorithm [34] is a differentially private version of the stochastic gradient descent algorithm commonly used to train neural networks. While differentially private learning has the advantage of having a formal mathematical definition, the $\epsilon$ and $\delta$ parameters can be difficult to choose and interpret. This issue is compounded by the fact that effective differential privacy typically works by adding noise to the aggregation (e.g., the training algorithm), which may hinder efficient training [35]. Furthermore, differential privacy was originally designed for database records, and some have argued that it is ill-suited to the unstructured nature of natural language [36].

In contrast to differential privacy, homomorphic encryption aims to protect the result of an input *X* and its output $M(X \mid D)$ rather than *D* (e.g., the data used to train a machine learning model *M*) itself. This is achieved by implementing *M* using operations that handle encrypted data, meaning that both *X* and $M(X \mid D)$ are knowable only to the person *using* the model [29]. Homomorphic encryption allows users to use a model owned by another party safely. The technique enables private inferences that do not disclose any information about the data to the owner of the model nor to any potential eavesdropper. However, it does not protect the *owner* of the model from attacks such as membership inference attacks or training data extraction attacks since the output of the inference is made available to the user initiating the inference.

With the growing availability of models capable of high-quality natural language generation, some have considered creating synthetic training data. This data, being synthetic, is assumed to be non-sensitive. By synthesizing data, the use of sensitive clinical data can be reduced [37] or done away with entirely [38, 39]. Synthetic data has been used in several studies to train well-performing fine-tuned clinical NLP models while limiting the risk of exposing private information from the original data [37–39]. There are fewer examples of models *pre-trained* using synthetic data. This is likely due to, at least in part, the computational costs of operating the large language models required to produce enough high-quality synthetic text. However, the example of GatorTronS [40] shows that this approach is indeed possible and that models pre-trained on synthetic text can perform well. On the other hand, the extent to which a synthetic text itself may contain sensitive data is poorly understood. The risk that the synthesizing language model accidentally generates parts of its own training data cannot be ruled out.

### Automatic de-identification and pseudonymization

Many of the aforementioned privacy-preserving techniques are not specific to natural language data. Differential privacy, for example, was originally designed for database-style structured data where each row is to be protected. Unstructured natural language data stands out as a particularly high-dimensional data form. In contrast to structured database rows, it can be difficult to exhaustively specify all of the information contained in an EHR. On the other hand, another feature of textual data is that many words or phrases can be replaced with similar information without changing the overarching meaning of a text. Examples of this phenomenon are synonyms which, broadly speaking, are interchangeable words that have the same meaning.

**Fig. 1:**
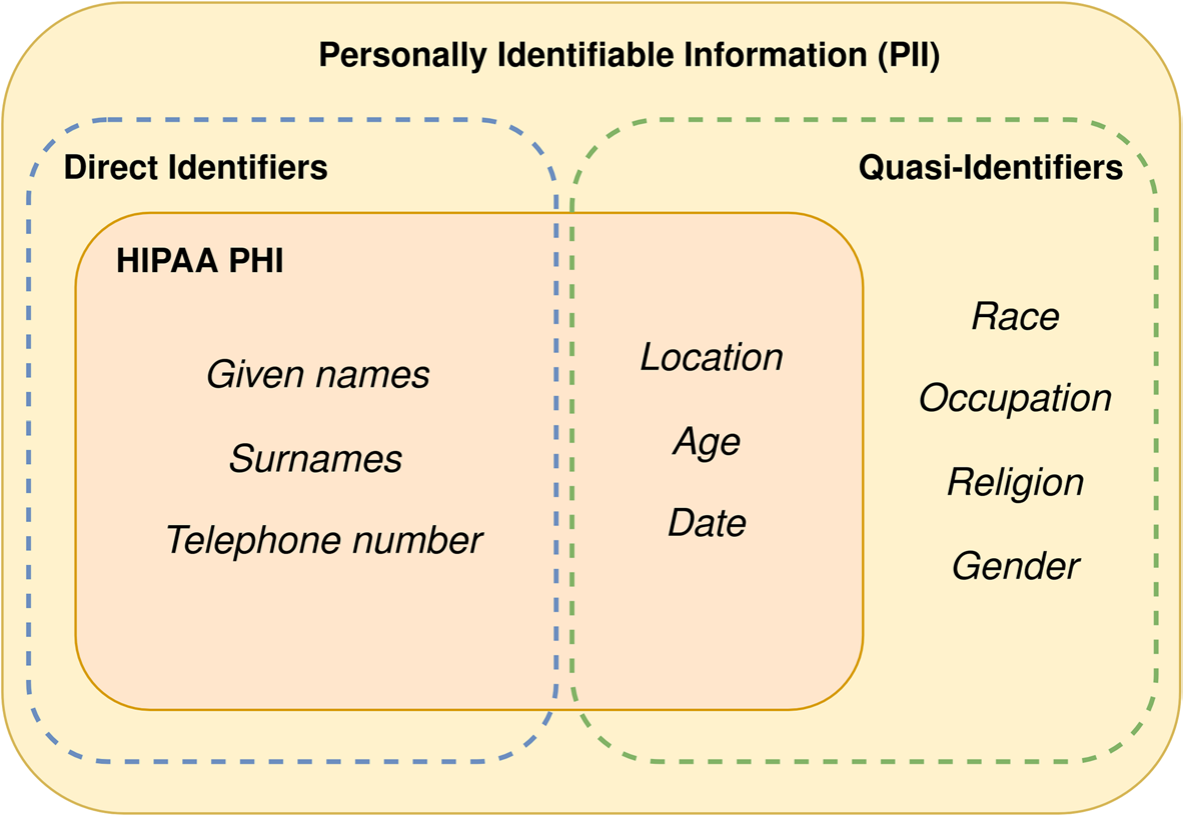
The HIPAA regulation in the United States lists 18 types of PII, called Protected Health Information (PHI), that should be removed for privacy reasons. These cover most of the PII types that are typically considered to be direct identifiers. However, as the figure illustrates, there are many quasi-identifiers that are not covered by this PHI definition

Automatic de-identification typically relies on NER to remove sensitive entities, such as data constituting personally identifiable information (PII). These entities usually cover direct identifiers such as names, but also cover *quasi-identifiers* such as locations, ages, and dates. Quasi-identifiers are PII that do not directly identify a person, but that may do so when combined with other quasi-identifiers or with auxiliary information. A commonly used set of PII is the collection of entities designated as personal health information (PHI) by the HIPAA regulation [41] in the United States. Examples of PII, PHI, and how they relate to different types of identifiers can be found in Fig. 1. In this article, we use the broader term PII. However, the set of PII covered by the de-identifiers is based on the PHI described by the HIPAA regulation [42].

**Fig. 2:**
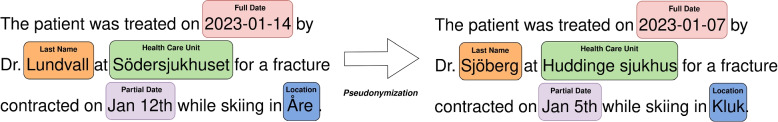
The pseudonymizers used in this study replace detected sensitive entities with realistic surrogates. The figure illustrates some of the entities considered by the system. The surrogate values are selected to preserve as much information as possible. However, an adversary with knowledge of Swedish geography would realize that, in this example, Kluk is an unlikely place to go skiing

De-identification is typically done in two main steps. First, the NER model of the de-identifier is used to detect entities that are PII. Next, these are *sanitized* in some way. Examples of sanitization techniques include replacing entities with their class name, masking them with a nondescript placeholder, and replacing them with surrogate values. This study focuses on the last strategy—*pseudonymization*—which replaces sensitive entities with realistic replacement values of the same entity type. These should preferably be chosen cleverly to preserve as much semantic information as possible without harming privacy. An example of how this process can work is illustrated in Fig. 2.

**Fig. 3:**
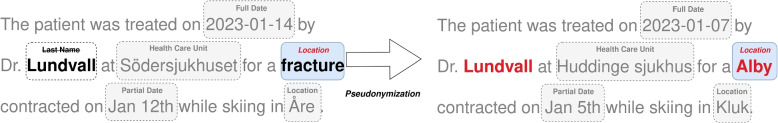
The NER models that power pseudonymizers are never perfect. When recall is insufficient, they will miss names such as *Lundvall*, which will remain exposed in the text. When there are problems with precision, non-sensitive words will be changed to irrelevant replacement values. In the worst case, a clinically relevant term like *fracture* may be replaced with a surrogate PII entity that harms data utility

The goal of pseudonymization is to remove the PII most likely to be used to re-identify individuals. However, it is important to recognize that pseudonymizers are never perfect. The NER models that power them often have imperfect recall and precision. Imperfect recall is a privacy issue since low recall implies that the model will miss sensitive entities that should be sanitized. On the other hand, low precision will result in many non-sensitive entities being replaced with inappropriate values. In the worst case, poor precision can lead to task-relevant words being replaced with irrelevant information, corrupting the datapoint and potentially having a negative impact on data utility. Both the low-recall and low-precision scenarios are illustrated in Fig. 3.

Pseudonymization is related to but different from *anonymization*. Although the terms are sometimes used interchangeably in the literature, anonymization is typically associated with stronger privacy guarantees. For example, when the term is used in the GDPR^1^ it is often understood as implying complete and irreversible removal of any information that can be used to partially or fully identify an individual [44]. Pseudonymization, as understood in this study, does not fulfill this stricter requirement. Rather, it is a process that *enhances* the privacy of data.

In contrast to other techniques within the field of privacy-preserving machine learning, pseudonymization is a text-specific technique for privacy preservation that harnesses the particular characteristics of natural language. When successfully applied, pseudonymization preserves the overall semantics of a datapoint while removing sensitive information. This scenario increases the privacy of a dataset while preserving its utility. However, when precision is not high enough, erroneous classifications and subsequent replacements will lead to a corruption of the data. The aim of this study is to demonstrate that, with a reasonably strong NER model, this does not happen often enough to harm the utility of the data for pre-training or fine-tuning clinical BERT models.

### Utility for machine learning using sanitized text

An early study on using pseudonymized EHRs is described by Yeniterzi et al. [45]. The authors trained NER models for detecting PII using both the pseudonymized and the original data. They found that the results deteriorated significantly when training on pseudonymized data and evaluating on unaltered text, with the F_1_ score falling from 0.862 to 0.728.

Lothritz et al. [23] study the impact of de-identification on a wide range of general-domain datasets. They employ a variety of sanitization strategies, including two pseudonymization strategies of different sophistication. They evaluate these strategies using ERNIE [46] and BERT models on eight different downstream tasks. Their results show that de-identification harms the utility of their datasets, but that this harm was small. The results also show that pseudonymization yields the strongest performance among the considered sanitization strategies.

Another study using sanitized text for machine learning is described by Berg et al. [47]. The authors pseudonymized Swedish clinical texts and then used them to train two different machine learning algorithms to detect PII. These algorithms were then evaluated on real Swedish clinical text data. The study aimed to enable sanitized training data to be transferred between hospitals for performing de-identification tasks. The authors tried two machine learning algorithms: conditional random fields (CRF) and long short-term memory (LSTM) networks. CRF gave the best results on training on sanitized text and de-identifying real clinical text; however, the performance on identifying several PII classes deteriorated, with the overall recall decreasing from 85% to 50%. This effect was primarily observed for the PII classes *Location*, *Health Care Units* and *Full Date*.

Berg et al. conducted another study [48] using four different strategies to sanitize the training data for downstream tasks, where models with different levels of recall were used to sanitize a set of Swedish datasets for clinical NER. Using a model with high recall is a good strategy in terms of privacy since it will identify more sensitive entities. However, these benefits may come at the expense of lower precision and more false positives. The study evaluated four different strategies for sanitizing the datasets: pseudonymization, masking the sensitive entities, replacing them with their class name, and removing the entire sentences in which sensitive entities were detected. The impact of sanitizing the data was evaluated by training CRF models for three clinical NER tasks using different sanitized datasets. Overall, the pseudonymization strategy had the smallest negative impact on the downstream tasks, while the sentence removal strategy resulted in a larger performance deterioration.

The overlap between PII and clinical entities is a source of potential harm to utility and has been thoroughly investigated by Berg et al. [48]. It was found that only one percent of clinical entities were affected by the de-identification process. The worst affected PII classes were *Health Care Unit* and *Person* (first and last name), which tended to overlap with the clinical entities *drug, body part, disorder* and *finding*. A later study [49] indicated that the risk of misclassifying eponyms (e.g., diseases like *Alzheimer disease* that are named after medical doctors) is lower when using BERT-based PII classifiers compared to earlier approaches. However, clinical entities are diverse, and there are other cases where misclassifications could be an issue.

Vakili et al. [22] evaluated the impact of pre-training BERT models using de-identified and unaltered data. Two sanitizing strategies were used: pseudonymization and sentence removal. Two models were adapted to the clinical domain by pre-training using clinical data sanitized with each strategy. The resulting models were then evaluated on six downstream tasks. The results showed no negative impact from pre-training using de-identified data compared to using unaltered data. Similarly, Vakili & Dalianis [20] evaluated the impact of fine-tuning a clinical BERT model using pseudonymized or unaltered datasets. They evaluated their approach using three downstream tasks, again finding no significant difference between training on unaltered or pseudonymized data. This study further builds upon the previous studies and provides deeper examinations of the interactions between pseudonymization and data utility. Furthermore, we demonstrate that pseudonymization can be applied both to the pre-training *and* fine-tuning data without harming the performance on clinical NLP tasks.

## Methods and materials

This study relies on a large number of datasets and models, mainly created using data from the Swedish Health Record Research Bank (Health Bank)^2^. The original data were collected from the Karolinska University Hospital [50] and consist of a large number of Swedish EHRs^3^. This section describes the data and models used in the experiments, and how these experiments were carried out.

### Clinical BERT models

**Fig. 4:**
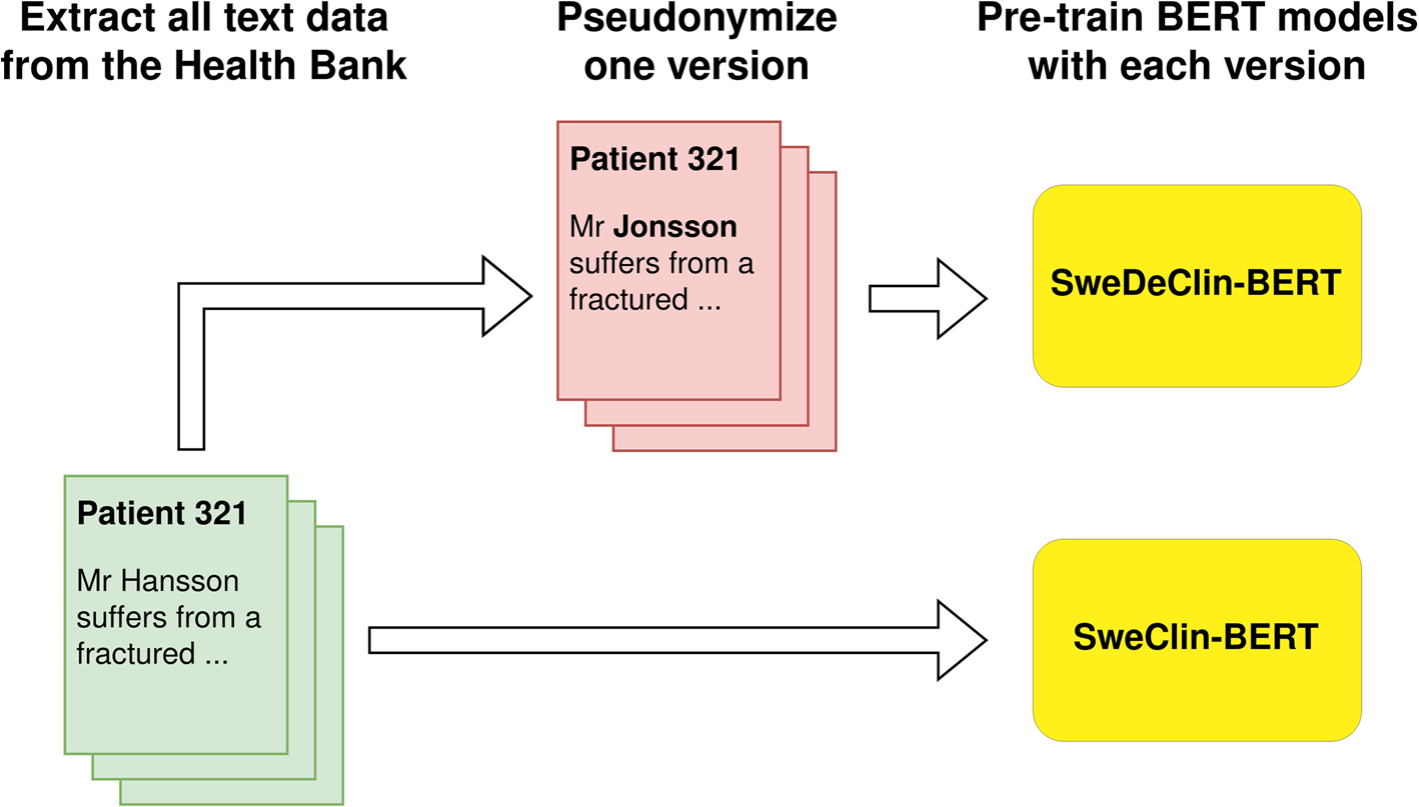
This study uses two different clinical BERT models created in earlier studies. SweClin-BERT is trained on a sensitive version of the Health Bank corpus [51], whereas SweDeClin-BERT is trained on a version that has been automatically pseudonymized [22]. Both models are initialized with the weights of KB-BERT [52]

This study examines the impact of pseudonymization applied to data for domain-adaptive pre-training and fine-tuning BERT models. As illustrated in Fig. 4, two different PLMs are used. One—*SweDeClin-BERT*—that has been trained using pseudonymized pre-training data [22], and another model—*SweClin-BERT*—that was trained on the unaltered version of the same dataset [51]. Both models were initialized using weights from the Swedish general-domain KB-BERT model [52] and were adapted to the clinical domain by pre-training for three epochs over the Health Bank corpus. Figure B1 in Appendix B contains a diagram showing how the models relate to other parts of the Health Bank.

The Health Bank corpus used for domain-adaptive pre-training consists of approximately 2.8 billion words which is comparable to the 3.3 billion words used to train KB-BERT [3]. We did not pre-train for more than three epochs for reasons of resource efficiency. This is justified by prior work using the same data [53] showing that longer pre-training was unnecessary when starting from a general-domain model.

### Five clinical downstream tasks

The utility of the models and datasets after and before pseudonymization was assessed using five clinical NLP tasks. The five tasks are based on corpora from the Health Bank infrastructure and are summarized^4^ in Table 1 and described in this section. The utility of each pseudonymization configuration was examined by measuring the performance of models fine-tuned on these tasks. Below is a list of the datasets as well as the abbreviated names used in Table 1 and other tables in the paper.

#### Stockholm EPR Gastro ICD-10 Corpus I (ICD-10)

The Gastro ICD-10 dataset consists of gastro-related discharge summaries and their assigned ICD-10 diagnosis codes. The discharge summaries relate to 4,985 unique patients. The ICD-10 codes are divided into 10 groups corresponding to different body parts; the ICD-10 codes range from K00 to K99. Each group contains several codes [55].

#### Stockholm EPR Clinical Entity Corpus (Clinical NER)

A clinical entity dataset encompassing 157,123 tokens and 20,675 annotated entities assigned to four clinical entity classes *Diagnosis*, *Findings*, *Body parts*, and *Drugs* [56]. The goal of the task is to identify and correctly label the clinical entities.

#### Stockholm EPR Diagnosis Factuality Corpus (Factuality NER)

A factuality diagnosis dataset specifying six levels of confidence regarding the factuality of a diagnosis. The dataset encompasses 6,865 annotated entities^5^ labeled as *Certainly Positive*, *Probably Positive*, *Possibly Positive*, *Possibly Negative*, *Probably Negative*, or *Certainly Negative* [57, 58]. The task consists of identifying tokens in the corpus specifying diagnoses and assigning them a factuality label.

#### Stockholm EPR Diagnosis Factuality Corpus (Factuality)

A dataset which is a variation of the *Stockholm EPR Diagnosis Factuality NER Corpus* that instead assigns a factuality level to the entire document. The classification task is a multi-label classification problem where the model needs to predict the factuality of each document. The labels are the same as in the NER version of the task.

#### Stockholm EPR ADE ICD-10 Corpus (ADE)

The ADE corpus contains 21,725 discharge summaries describing adverse drug events (ADEs). The task is a binary classification task, where positive samples have been assigned an ICD-10 code that denotes an ADE. Negative text samples in each group have been assigned an ICD-10 code describing a diagnosis that is not drug-induced. The task is to determine whether the diagnosis defined by the ICD-10 code was induced by an ADE or not [22].

**Table 1:** The five tasks were based on four different clinical corpora from the Health Bank. This table lists the size of each corpus in terms of the number of documents and tokens. The table also specifies the number of possible classes and whether the tasks are document-level or token-level classification tasks

Corpus	Documents	Tokens	Classes	Level
*ICD-10*	6,062	930,550	10	Document
*ADE*	21,725	931,778	2	Document
*Factuality*	3,710	102,223	6	Document
*Factuality NER*	3,822	286,205	6	Token
*Clinical NER*	3,120	178,672	4	Token

### Pseudonymization

The pseudonymization performed in this study relies on NER to locate sensitive entities that should be replaced. Two such NER models are used. Both are based on BERT and are fine-tuned on the Stockholm EPR PHI Corpus [42]. This corpus contains 380,000 tokens and 4,480 manually annotated entities in nine classes based on the American HIPAA regulation. One model *pseudo+* uses a non-pseudonymized Swedish clinical BERT model [59] and another, slightly weaker model called *pseudo* is based on SweDeClin-BERT [22]. Tables 2 and 3 list the per-class performance of both NER models as measured using the test splits of their training data. Figure B2 in Appendix B shows how these models relate to other parts of the Health Bank.

Two pseudonymized versions of each dataset described in the previous section were created, one for each NER model. Sensitive entities were detected and then replaced with realistic surrogate values based on the method described in this section. The number of sensitive entities detected by the pseudonymizers is displayed in Tables 4 and 5. These numbers include both false and true positives and indicate the degree to which the data were altered in the pseudonymization process.

An overview of the algorithm for surrogate selection is available in Dalianis [60], which describes the first version of the pseudonymizer. The system has been further refined since its initial conception. One adaption made from the original pseudonymizer is that the name lists used to replace first and last names have been expanded to include a wider range of names. The original system only considered the most common Swedish names, while the current system chooses from 244,000 first names and 34,000 surnames. However, a limitation of the pseudonymizer is that it lacks functionality for replacing organizations. As shown in Tables 4 and 5, organizations are very infrequent, meaning that the privacy and performance implications are limited.

The pseudonymizer created by Dalianis [60] replaces many entities using word lists. For example, a gendered name is replaced with another name typically associated with the same gender, and a gender-neutral name is replaced with a gender-neutral name. Streets and places in Stockholm randomly with other streets in Stockholm. Similarly, other locations in Sweden are replaced with locations in the same county, and similar logic exists to replace country names with names of countries in the same continent. Health care units are changed to other health care units using a list of known clinics. Other entities are changed using rules. Postal codes are replaced with more common postal codes with large populations. Dates are shifted one or two weeks earlier or later. Years and ages are handled similarly and are increased or decreased by a small and random number of years. Phone numbers are changed to other phone numbers according to the formatting rules for Swedish phone numbers.

**Table 2:** The recall and precision of the *pseudo+* model for each PII type are displayed. The model is a clinical BERT model [59] that has been fine-tuned and evaluated using the *Stockholm EPR PHI Corpus* [42]

PII Class	Recall	Precision
*Age*	100%	100%
*First Name*	100%	100%
*Last Name*	98%	98%
*Partial Date*	99%	97%
*Full Date*	90%	91%
*Phone Number*	81%	68%
*Health Care Unit*	85%	94%
*Location*	100%	100%
*Organization*	71%	100%

**Table 3:** The recall and precision of the *pseudo* model for each PII type are displayed. The model is based on the pseudonymized SweDeClin-BERT model and has been fine-tuned and evaluated using the *Stockholm EPR PHI Corpus* [42]

PII Class	Recall	Precision
*Age*	100%	100%
*First Name*	97%	98%
*Last Name*	96%	97%
*Partial Date*	99%	98%
*Full Date*	87%	91%
*Phone Number*	93%	89%
*Health Care Unit*	89%	88%
*Location*	89%	81%
*Organization*	29%	80%

**Table 4:** Sensitive entities detected by the *pseudo* model

PII Class	Factuality NER	Clinical NER	ICD-10	Factuality	ADE
*Age*	1,392	1,149	3,060	1,353	2,995
*First Name*	528	274	1,185	510	3,965
*Last Name*	1,105	274	1,829	1,062	4,257
*Partial Date*	681	554	11,371	644	4,305
*Full Date*	128	137	18,875	125	22,296
*Phone Number*	148	45	141	142	460
*Health Care Unit*	3,554	2,005	3,365	3,406	7,635
*Location*	110	78	510	105	689
*Organization*	5	1	37	4	59
***Total words***	*253,124*	*191,202*	*798,120*	*239,722*	*788,930*

**Table 5:** Sensitive entities detected by the *pseudo+* model

PII Class	Factuality NER	Clinical NER	ICD-10	Factuality	ADE
*Age*	955	764	2,565	929	2,257
*First Name*	523	283	1,378	506	3,884
*Last Name*	1,055	707	1,904	1,016	4,064
*Partial Date*	369	316	5,740	355	2,995
*Full Date*	110	121	12,703	107	17,552
*Phone Number*	118	39	75	113	172
*Health Care Unit*	4,285	2,282	12,654	4,117	9,751
*Location*	182	102	1,217	172	985
*Organization*	4	12	6	1	66
***Total words***	*253,124*	*191,202*	*798,120*	*239,722*	*788,930*

### Evaluating the impact of pseudonymization

As previously discussed, pseudonymization often entails a certain degree of data corruption. The main experiment in this study examines this effect on the downstream performance of clinical BERT models pre-trained and fine-tuned on pseudonymized clinical training data.

**Fig. 5:**
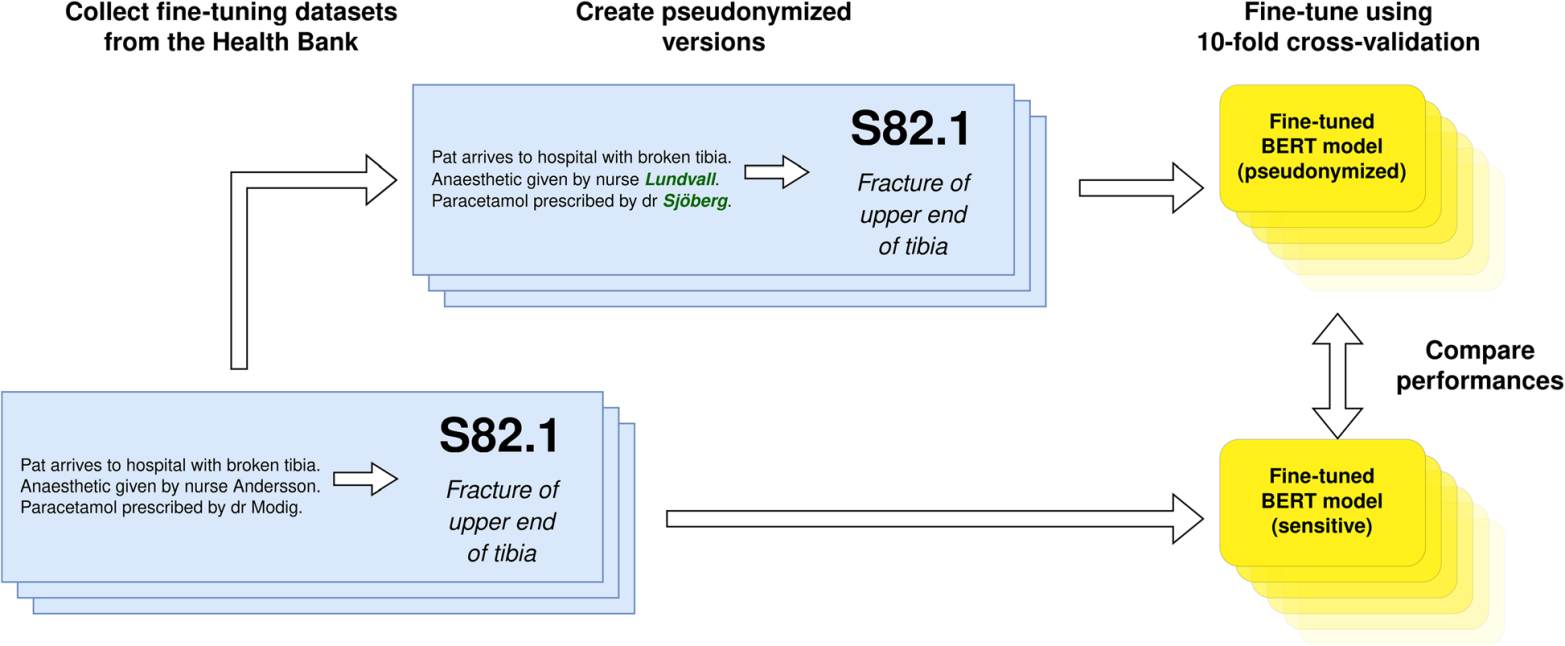
Every dataset described in the “Five clinical downstream tasks” section was pseudonymized using both the *pseudo* and *pseudo+* de-identifiers. SweDeClin-BERT and SweClin-BERT were fine-tuned using the non-pseudonymized and the two pseudonymized versions of the datasets. All models were compared based on the F_1_ scores aggregated from the 10-fold cross-validation of each model

Once the datasets for the five clinical downstream tasks had been pseudonymized, a series of evaluations were carried out. Each version of every dataset was used to fine-tune and test both BERT models using 10-fold cross-validation [61], as illustrated in Fig. 5. Since the pseudonymization procedure is a deterministic pre-processing step, the pseudonymized models are tested on pseudonymized folds. The repeated training and evaluation using different splits resulted in a range of evaluation metrics used to estimate the mean and standard deviation of each configuration. The configurations were compared based on their F_1_ scores^6^ [63]. All fine-tuning configurations ran for a maximum of 10 epochs, with early stopping implemented to avoid overfitting and unnecessary computations.

In total, 30 different combinations of models and datasets were evaluated using 10-fold cross-validation. For every downstream task, we compare the difference in the performance of all combinations of models and pseudonymization approaches. The difference between each pair was tested for statistical significance using a *Mann-Whitney U test*^7^ [64, 65] by comparing the F_1_ scores of every fold in both models’ 10-fold cross-validations.

## Results

The 30 different model-dataset configurations combined with the 10-fold cross-validation resulted in 300 fine-tuned models. The evaluations of these models were used to produce F_1_ for each configuration and downstream task. The means and standard deviations of each evaluation are listed in Table 6. From studying the columns of the table, it is apparent that most of the values are within a standard deviation of each other.

**Table 6:** The table compares the performance of each combination of models and datasets. The scores are the mean F_1_ scores together with their standard deviation based on the results from the 10 folds. **P** stands for pre-training data and **F** for fine-tuning data. A ✗ denotes that no pseudonymization was done, a ✓ that it was done using the *pseudo* model and a **+** means that pseudonymization was performed using the *pseudo+* model

Pseudonymized		Factuality	Clinical Entity	ICD-10	Factuality	ADE
P	F	NER	NER	Classification	Classification	Classification
✗	✗	*0.686±0.013*	*0.851±0.012*	*0.821±0.012*	*0.729±0.020*	*0.186±0.009*
✗	✓	0.639±0.038	0.843±0.011	0.810±0.011	0.725±0.021	0.190±0.017
✗	**+**	0.668±0.024	0.841±0.011	0.814±0.008	0.726±0.018	0.188±0.014
✓	✗	0.696±0.019	0.861±0.011	0.835±0.010	0.726±0.025	0.188±0.011
✓	✓	0.663±0.048	0.856±0.009	0.825±0.010	0.716±0.016	0.198±0.013
✓	**+**	0.695±0.013	0.853±0.011	0.832±0.007	0.733±0.022	0.205±0.018

Comparing every configuration within each downstream task resulted in 150 Mann-Whitney U tests being performed. Out of these, 126 comparisons showed no statistically significant difference for $p<0.05$. The remaining 24 comparisons showed varying degrees of statistical significance. To facilitate a focused analysis of the results, a curated sample of the significant results is listed in Table 7. These are limited to the cases where using real data outperformed using pseudonymized data, as these examples challenge the main hypothesis of the study. The full list of the 24 statistically significant differences is provided in Table A1 of Appendix A.

**Table 7:** Out of 24 statistically significant results, 11 are cases where using non-pseudonymized data yields better results than using pseudonymized data. All of these find this effect with regard to the fine-tuning data. The *p-value* is the result of the Mann-Whitney U test for determining if the *Weaker model* performs worse than the *Stronger model*. For each model, **P** indicates whether the pre-training data was pseudonymized, and **F** indicates if the fine-tuning data was pseudonymized. Again, a ✗ denotes that no pseudonymization was done, a ✓ that it was done using the *pseudo* model and a **+** means that pseudonymization was performed using the *pseudo+* model

Row	Task	Weaker model		Stronger model		*p*-value
		P	F	P	F	
(1)	ICD-10	✗	✓	✗	✗	0.0378
(2)	Factuality NER	✗	✓	✗	✗	0.0014
(3)	Clinical NER	✗	**+**	✗	✗	0.0269
(4)	ICD-10	✗	✓	✓	✗	0.0007
(5)	Clinical NER	✗	✓	✓	✗	0.0029
(6)	Factuality NER	✗	✓	✓	✗	0.0005
(7)	ICD-10	✗	**+**	✓	✗	0.0011
(8)	Clinical NER	✗	**+**	✓	✗	0.0022
(9)	Factuality NER	✗	**+**	✓	✗	0.0156
(10)	ICD-10	✗	✗	✓	✗	0.0086
(11)	Clinical NER	✗	✗	✓	✗	0.0226

Notably, none of the statistically significant differences were cases where SweClin-BERT outperformed SweDeClin-BERT. This is apparent from the *P* column for the *Weaker model* only containing ✗’s. This implies that SweDeClin-BERT is a stronger model for the downstream tasks in this study. In that case, this difference in general model performance explains rows 4–11 of Table 7. Furthermore, there are no examples where training SweDeClin-BERT on different forms of fine-tuning data yielded statistically significant differences in performance.

The first three rows in Table 7 show that training SweClin-BERT using non-pseudonymized data sometimes yields statistically significant improvements compared to using pseudonymized data. This is found for one pair of configurations for three of the tasks. There are no examples where training on real data outperforms both forms of pseudonymized data. For example, the first row finds a statistically significant improvement from using real ICD-10 data rather than data pseudonymized using the *pseudo* model, but no significant difference is found if the *pseudo+* model is used.

## Discussion

The previous section presents several interesting findings. In this section, the results of the study are analyzed and contextualized. We also provide ideas for future work and discuss the limitations of our study.

### Interpreting the significant results

The results of this study are based on a large number of Whitney-Mann U tests. When performing 150 statistical tests, there is a non-trivial risk of finding spurious statistical differences. The standard cut-off of $p < 0.05$ still risks finding differences by chance 1 out of 20 times. Nevertheless, there are some trends in Table 7 that are interesting to discuss.

First, it is notable that none of the statistically significant comparisons find that pre-training with real data outperforms pre-training with pseudonymized data. A similar result was indicated in a previous study by Vakili et al. [22]. However, it is important to note that only two pre-trained models were compared in this study. While the results strongly suggest that SweDeClin-BERT is better than SweClin-BERT, this does not mean that pre-training with pseudonymized data is better *in general*. Examining this would require pre-training many more BERT models with and without pseudonymizing the data. It would likely also require comparing pre-trained models initialized from random weights rather than the weights of a general-domain model. While this could be interesting to study, it is beyond the computational constraints imposed by the scope of this study.

Some of the statistically significant results in Table 7 do indicate that fine-tuning a non-pseudonymized model using unaltered data can yield stronger results than fine-tuning with pseudonymized data. However, this is only found for three of the five downstream tasks. Furthermore, none of these results hold for *both* of the pseudonymizers. The results in Table 6 also show that these examples are still within a standard deviation of each other. The results where fine-tuning on real data *does* outperform using both pseudonymized data (such as rows 4 and 7 of Table 7) are results where SweDeClin-BERT outperforms SweClin-BERT. Thus, these cases are better explained by the overall stronger results of SweDeClin-BERT. Crucially, for the purposes of this study, there are *no* examples of statistically significant differences where a model trained using end-to-end pseudonymization is outperformed by a non-pseudonymized version. The hypothesis of this study holds since we find no evidence of any significant deterioration from pre-training and fine-tuning using automatically pseudonymized data.

### Quantifying privacy benefits

An important limitation of this study is that the privacy benefits of pseudonymization are only quantified in terms of the number of removed sensitive entities. This assumes that the sensitivity of the training data directly corresponds to the sensitivity of the model. This assumption may be pessimistic since it is unlikely that the trained model will memorize all remaining sensitive entities. On the other hand, relying on metrics such as recall and precision also obscures any particularities in the *specific* entities that are missed and if these could be more at risk of memorization.

Previous research has suggested that *membership inference attacks* can be used for estimating the degree of memorization in a model [30, 31, 66]. This approach can be effective for some privacy-preserving techniques, such as differentially private learning [34]. Unfortunately, this method has been shown to work poorly when applied to models trained using pseudonymized data [67].

The lack of robust methods for quantifying the privacy gains of pseudonymizing training data remains a significant drawback of the technique. For example, differentially private learning, as described in the background, gives rigorous mathematical privacy guarantees. In contrast, while the results in this article show that privacy can be gained without sacrificing data utility, the exact privacy gains remain unknown. However, the estimated amount of remaining PII in the training data provides an upper bound concerning the entities covered by the pseudonymizer. In any case, there is no consensus on how privacy *should* be measured from a regulatory standpoint. Indeed, according to some strict but prominent interpretations of the GDPR, legal use of data containing PII may be next to impossible [44]. The development of GDPR-compliant privacy metrics should preferably be conducted in communication with the legal community.

### Domain-adaptive pre-training

Both pre-trained models—SweDeClin-BERT and SweClin-BERT—are initialized with the weights of the general-domain Swedish KB-BERT model. As shown by Lamproudis et al. [53], this allows them to converge faster when compared to pre-training from randomly initialized weights. This is beneficial from a resource perspective, as pre-training is both time and energy consuming. It can also have positive benefits for privacy, as the models have been trained using both sensitive and non-sensitive corpora.

While there are benefits to initializing the models from an already capable general-domain model, this decision is also a possible limitation of our methodology. While the previous study by Lamproudis et al. showed that domain-adapted models and models pre-trained from scratch eventually converge, they did not look at whether pseudonymization may affect this result. Although PII constitutes a very small portion of the total data [68], it is plausible that pseudonymization introduces variability to the pre-training corpora. This added variability could make it easier or harder to learn. Whether pseudonymizing the pre-training corpora has any substantial impact on the rate of convergence or the final quality of a model trained from scratch is an interesting idea for future research.

### Identifying PII in clinical text

The effectiveness of end-to-end pseudonymization as a privacy-preserving technique depends largely on the ability to accurately identify PII in the corpora used for pre-training and fine-tuning the clinical language models. In this study, a manually annotated PII corpus [42] was used to fine-tune clinical BERT models to identify PII. The performance of these models – estimated through evaluations on held-out test data from the *Stockholm EPR PHI Corpus* – is reported in Table 2 and 3. While both precision and recall are fairly high for most PII classes, we have not evaluated the performance of the model to identify PII in the downstream task corpora, nor in the pre-training corpus. A previous study showed that the performance of a CRF model trained on this PII corpus performed worse when applied to other types of clinical notes and that the performance varied quite considerably across different types of clinical notes, i.e. produced in different clinical specialties, written by persons in different professional roles, and under different headings [69]. In part, this may also be explained by the fact that the prevalence of PII varies across different types of clinical notes. While the overall PII density^8^ was estimated to be around 1.57%, it was estimated to be as low as 0.97% for notes written by physiotherapists and as high as 2.19% in discharge notes [68].

The results of this study show that the utility of the models was not negatively affected by being trained on pseudonymized data compared to using the original sensitive data, allowing privacy risks to be reduced without sacrificing predictive performance. However, the utility would likely, at some point, be reduced if a pseudonymization system with poor precision substantially distorted the data. Here, two pseudonymizers with different performance levels were evaluated and the results did not indicate any significant difference in terms of their impact on data utility for fine-tuning clinical BERT models. However, previous work evaluating the impact of pseudonymization on the performance of clinical NER tasks showed that training pseudonymizers with higher recall at the expense of lower precision does eventually harm data utility [48]. In future work, it would be interesting to determine at what point – e.g., at a certain level of precision – that data utility starts to be significantly impacted. However, this tolerance threshold would likely need to be determined separately for different downstream tasks.

### Sharing data and models

The clinical language model SweDeClin-BERT and the Stockholm EPR Gastro ICD-10 Pseudo Corpus are available for academic use worldwide^9^. Based on the results of this study, we plan to make the other pseudonymized corpora used in this study available as well. However, this requires supplementary ethical approval from the Swedish Ethical Review Authority. Moving forward, an interesting issue is whether it is also possible to make these pseudonymized clinical corpora and language models available to industry. This would enable commercial applications that could be used in real clinical settings. The benefits of sharing data and models must also be balanced against the privacy risks of doing so. From a legal standpoint, sharing data among academics can be justified due to the explicit provisions that the GDPR makes for research. These provisions do not apply to commercial use, making sharing data with commercial partners difficult.

As noted earlier in the discussion, there is no consensus regarding how privacy should be quantified when dealing with NLP models. The current flora of PLMs is heterogeneous, including both masked language models like BERT and generative models such as the GPT family. Risk assessments should likely be done on a per-model basis, given the vast differences between models in terms of architectures, the scale of their pre-training data, their number of parameters, and what privacy-preserving techniques have been applied. The models used in this study are based on the modestly-sized BERT_BASE_ model, a non-generative model composed of approximately 110 million parameters. Although there have been several studies on the matter [24–27], there are no known examples of successful training data extraction attacks targeting BERT models.

It is important to note that the performance measures attained in this study do not necessarily hold for other sets of hospitals. All models and datasets use data from the Health Bank research infrastructure, which come from a specific set of medical clinical units. It is well-known that models trained on one set of data sources may perform worse when confronted with novel data [37]. Indeed, as noted in the previous section, performance can vary even within a set of data sources. Further complicating the situation, the clinical domain generally struggles with the many restrictions on sharing data. While understandable and justified from a privacy perspective, these restrictions make it difficult to evaluate models and datasets cross-institutionally. Nevertheless, two studies applying SweDeClin-BERT to new data have been carried out [70, 71], with encouraging results.

## Conclusion

This study evaluates the impact of pre-training and fine-tuning using automatically pseudonymized training data. Two clinical BERT models, one trained on real data and one trained on pseudonymized data, are evaluated on five clinical downstream tasks. The datasets for these tasks are used both in unaltered form and in pseudonymized versions. The results from evaluating all different configurations of models and datasets are tested using Mann-Whitney U tests.

The analysis of the statistically significant tests finds limited evidence supporting that, in some cases, fine-tuning non-pseudonymized PLMs may work better if using non-pseudonymized data. Such an effect, if real, is small. Furthermore, we find no cases where pre-training and fine-tuning using pseudonymized data end-to-end harms utility. This demonstrates that pseudonymization can decrease the privacy risks of using clinical data for NLP without harming the utility of the machine learning models.

### Supplementary Information

Supplementary Material 1.

## Data Availability

The datasets generated and analyzed during the current study are not publicly available due to legal and ethical privacy concerns, as discussed in the section on “Sharing data and models”. The pseudonymized versions of the ICD-10 corpus as well as the pre-trained SweDeClin-BERT model, are available from the corresponding author on reasonable request.

## Footnotes

1 The GDPR is the General Data Protection Regulation of that is applied throughout the European Union [43].

2
http://www.dsv.su.se/healthbank

3 This research has been approved by the Swedish Ethical Review Authority under permission no. 2019-05679.

4 The number of tokens was calculated using the Punkt tokenizer for Swedish in NLTK [54].

5 The dataset also contains 199 entities annotated for purposes irrelevant to our experiments. These annotations were ignored.

6 Metrics for the token classification tasks were calculated using the seqeval library [62] in strict mode.

7 This test is also sometimes referred to as a Wilcoxon rank-sum test.

8 Defined as the number of PII-labeled tokens divided by the total number of tokens.

9 Contact the authors for details on how to gain access to the data and models.
