# Supplementary material for: End-to-end pseudonymization of fine-tuned clinical BERT models: Privacy preservation with maintained data utility
Source: BMC Med Inform Decis Mak. 2024 Jun 12;24:162. doi: 10.1186/s12911-024-02546-8 (PMC11197357; doi:10.1186/s12911-024-02546-8)
Supplement: Supplementary file 1 — Supplementary Material 1. [file 12911_2024_2546_MOESM1_ESM.pdf]

Appendix A All Significant Results

Table 6 in Section 4 only included the statistically relevant results relevant to the analysis and discussion. Table A1 lists *all 24* statistically significant results obtained from the 150 comparisons that were made.

Task	Weaker model		Stronger model		p-value
	P	F	P	F	
ICD-10	✗	✓	✗	✗	0.0378
Factuality NER	✗	✓	✗	✗	0.0014
ICD-10	✗	✓	✓	✓	0.0014
Clinical NER	✗	✓	✓	✓	0.0070
ICD-10	✗	✓	✓	+	0.0002
Clinical NER	✗	✓	✓	+	0.0320
Factuality NER	✗	✓	✓	+	0.0002
ICD-10	✗	✓	✓	✗	0.0007
Clinical NER	✗	✓	✓	✗	0.0029
Factuality NER	✗	✓	✓	✗	0.0005
Clinical NER	✗	+	✗	✗	0.0269
ICD-10	✗	+	✓	✓	0.0057
ADE Classification	✗	+	✓	✓	0.0320
Clinical NER	✗	+	✓	✓	0.0029
ICD-10	✗	+	✓	+	0.0006
ADE Classification	✗	+	✓	+	0.0481
Clinical NER	✗	+	✓	+	0.0129
Factuality NER	✗	+	✓	+	0.0188
ICD-10	✗	+	✓	✗	0.0011
Clinical NER	✗	+	✓	✗	0.0023
Factuality NER	✗	+	✓	✗	0.0156
ADE Classification	✗	✗	✓	✓	0.0078
ICD-10	✗	✗	✓	✗	0.0086
Clinical NER	✗	✗	✓	✗	0.0226

Table A1 All 24 statistically significant results are listed alongside the task, the model configurations and the *p-values*. Similarly to Table 5 and Table 6, **P** denotes whether pre-training was done using pseudonymized data, and **F** if fine-tuning was done using pseudonymized data. As in the previously mentioned tables, a ✗ denotes that no pseudonymization was done, a ✓ that it was done using the *pseudo* model and a + means that pseudonymization was performed using the *pseudo+* model.

Appendix B Data Diagrams

The datasets and models used in this study relate to each other in complicated ways. As a supplement to the figures and descriptions in the main text, we provide some more detailed diagrams of the relevant data flows. Figure B1 shows what data was used to train the BERT models described in Section 3.1. Figure B2 shows which data and models were used to create the *pseudo+* and *pseudo* PII NER models. These are the NER models that were used to de-identify the downstream tasks, as described in

Section 3.3. Note that most of the steps illustrated in the figures are from prior work (indicated with references).

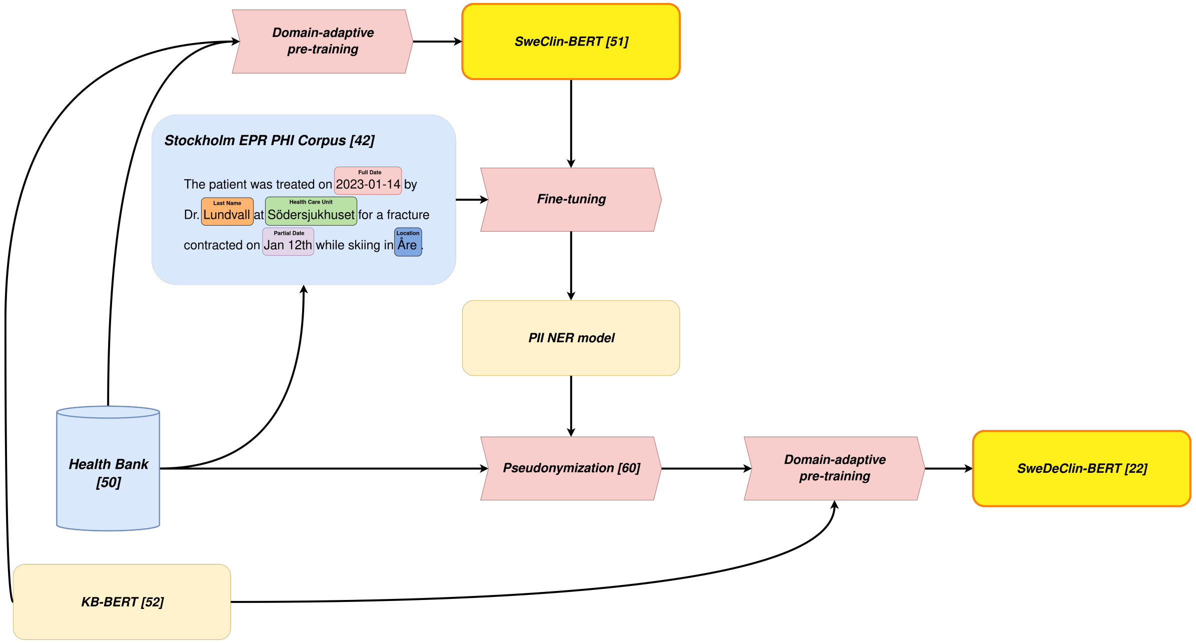

Fig. B1 The SweClin-BERT and SweDeClin-BERT models are both initialized from KB-BERT and the in-domain pre-trained using the Health Bank corpus. The difference is the SweDeClin-BERT was trained on a version which was automatically pseudonymized.

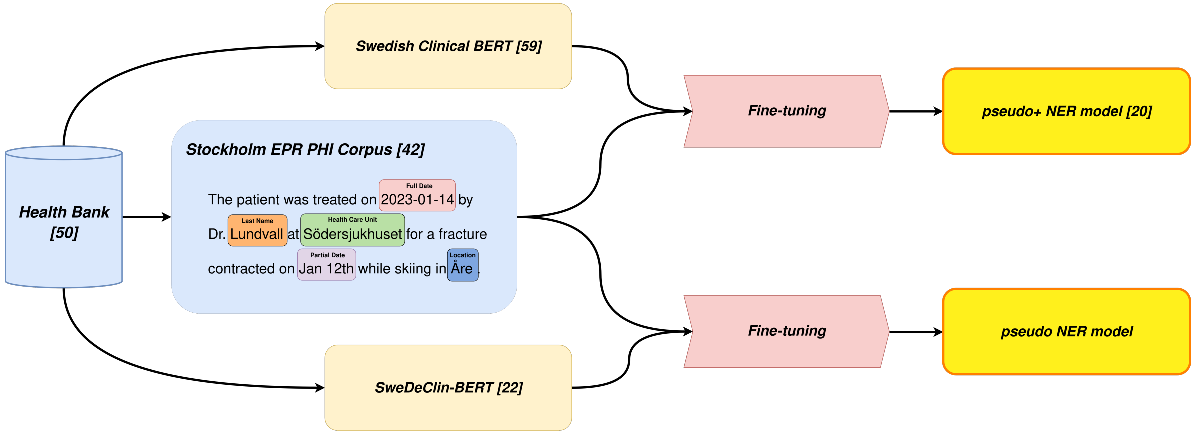

Fig. B2 The pseudonymization in this study relies on two NER models trained to detect PII. These differ in which BERT model was fine-tuned.

Appendix C Fine-Tuning Parameters

The study relies on multiple fine-tuned models. These models are used for de-identification and for evaluating the utility of the de-identified data. Tables C2 and C3 list the parameters used to fine-tune the models. The values are based on prior work.

Parameter	Value
Learning rate	0.00003
Batch size	64
Max. epochs	10
Optimizer	Adam
Scheduler	Constant
Early stopping?	Yes

Table C2 Training parameters set when training the *pseudo* and *pseudo+* models and when fine-tuning Clinical NER, Factuality NER, and ADE.

Parameter	Value
Learning rate	0.00002
Batch size	64
Max. epochs	10
Optimizer	Adam with weight decay
Weight decay	0.01
Excluded from weight decay	bias, layer norm
Scheduler	Linear with warmup
Warm-up	155 steps
Early stopping?	Yes
Patience	1
Threshold	0.5

Table C3 Training parameters set when training the ICD-10 and Factuality models.
